# Development and validation of prognostic model to predict mortality among cirrhotic patients with acute variceal bleeding: A retrospective study

**DOI:** 10.1002/jgh3.12550

**Published:** 2021-05-05

**Authors:** Sakkarin Chirapongsathorn, Kuntapon Akkarachinores, Amnart Chaiprasert

**Affiliations:** ^1^ Division of Gastroenterology and Hepatology, Department of Medicine Phramongkutklao Hospital and College of Medicine Bangkok 10400 Thailand; ^2^ Division of Nephrology, Department of Medicine Phramongkutklao Hospital and College of Medicine Bangkok 10400 Thailand

**Keywords:** cirrhosis, varices, variceal bleeding

## Abstract

**Background and Aim:**

Acute variceal bleeding (AVB) is a serious complication associated with high mortality. The aim of our study was to investigate mortality predictors and to develop a new simplified prognostic model among cirrhotic patients with AVB.

**Methods:**

A simplified prognostic model was developed using multiple logistic regression after identifying significant predictors of 6‐week mortality.

**Results:**

A total of 713 consecutive patients with AVB were enrolled. The 6‐week overall mortality rate was 18%. Multivariate analysis showed that shock, model for end‐stage liver disease (MELD) score, high‐risk stigmata of esophageal varices on endoscopic finding, and Glasgow Blatchford score were independent predictors of mortality. A new logistic model using these variables was developed. This model (cutoff value ≥ 4) area under the receiver operating characteristics (AUROC) was 0.93 and significantly higher than that of MELD score alone (0.74). Two validation analyses showed that the AUROC of our model was consistently high. The 6‐week rebleeding rate was 25.3%. Multivariate analysis showed that MELD score, Glasgow Blatchford score, history of upper GI bleeding, shock, and alcohol use were independent predictors of rebleeding.

**Conclusion:**

Our new simplified model accurately and consistently predicted 6‐week mortality among patients with AVB using objective variables measured at admission. Patients with higher MELD scores should be closely monitored due to the higher probability of 6‐week rebleeding.

## Graphical abstract

Our new simplified model accurately and consistently predicted 6‐week mortality among cirrhotic patients with acute variceal bleeding using objective variables measured at admission. Patients with higher model for end‐stage liver disease scores should be closely monitored due to the higher probability of 6‐week rebleeding.

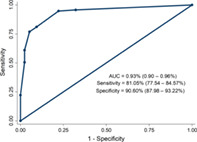

## Introduction

Patients with cirrhosis are at risk for developing complications including critical conditions such as acute variceal bleeding (AVB), sepsis, or hepatorenal syndrome.^1^, ^2^ AVB is a major life‐threatening complication and common among patients with cirrhosis.^1^ Current guidelines recommend endoscopic band ligation therapy for treatment of esophageal varices or glue injection/sclerotherapy for treatment of gastric varices combined with prompt vasoactive drugs and prophylactic antibiotic administration as the mainstream for treating AVB.^1^, ^3^ However, mortality remains high among patients with AVB.^4^, ^5^ Several prognostic models have been developed to predict prognosis of upper gastrointestinal bleeding (UGIB). Rockall and Glasgow Blatchford score are widely used in UGIB but mostly validated for predicting clinical outcomes for non‐variceal UGIB (NVB) and are poor at predicting prognostic outcomes among patients with AVB.^6^

Child–Pugh score and model for end‐stage liver disease (MELD) score are widely used to predict prognosis among patients with cirrhosis.^7^, ^8^ However, these models were not developed to predict prognosis of patients with AVB and so might be not applied to patients with AVB. Patients with AVB may develop others complications of cirrhosis and are at increased risk of developing bacterial septicemia and circulatory dysfunction that may lead to developing acute‐on‐chronic liver failure.^9^, ^10^ The aim of our study was to develop a simple prognostic model based on initial objective components among patients with AVB.

## Methods

### Study cohort and data collection

We collected administrative databases including all patients admitted to Phramongkutklao Hospital (Bangkok, Thailand) and Maharat Nakhon Sri Thammarat Hospital (Nakhon Sri Thammarat, Thailand) for portal hypertension‐related bleeding from October 2012 to September 2018. This study was approved by the Institutional Review Board Royal Thai Army Medical Department and permission was granted to use data from the head director of the hospitals. All data were gathered in the context of standard practice from clinical records of the patients, and were anonymized and collected in a protected database. All methods were carried out in accordance with relevant guidelines and regulations. No specific procedures were conducted for the study and informed consent was not required. Institutional Review Board Royal Thai Army Medical Department approved to waive the need for informed consent.

Patients with cirrhosis and acute bleeding from variceal bleeding including esophageal varices and gastric varices were considered eligible for the study. The diagnosis of cirrhosis was based on previous clinical history, liver biopsy, clinical data, and compatible findings on imaging including computer tomography and magnetic resonance imaging. Variceal bleeding was confirmed by esophagogastroduodenoscopy. Baseline clinical characteristics including shock, biochemical profiles, endoscopic findings, and imaging data of patients were recorded. Shock was defined as mean arterial pressure (MAP) <50 mmHg. This definition was chosen to confirm clinical syndrome of shock, which is not hypotension (MAP < 65 mmHg).

### Therapeutic interventions and definitions

All patients in the database were treated with standard care according to Baveno consensus workshops.^11^ All patients received octreotide as vasoactive agents, blood transfusion, and prophylaxis antibiotic drugs with ceftriaxone (91%) or cefotaxime (9%) from admission. Endoscopic band ligation within admission and Sengstaken‐Blakemore balloon tamponade rescue therapy was applied when necessary such as uncontrolled bleeding or rebleeding (rescue transjugular intrahepatic portosystemic shunt was unavailable in our centers). We combined secondary prophylaxis including β‐blockers at day 5 after index admission. The primary outcome analyzed in this study was 6‐week mortality according to the expanding consensus in portal hypertension report of the Baveno VI consensus workshop.^11^ The secondary outcomes analyzed in this study were 5‐day mortality and rebleeding.

### Prognostic models to predict mortality

We built prognostic model estimations of 6‐week mortality in AVB. We also evaluated the performance of the MELD and Glasgow Blatchford scores to determine prognosis of patients with AVB in our database. Our selected prognostic model was selected for cross validation.

### Statistical analysis

Descriptive statistics were used to characterize the demographic features of the study population. Continuous variables were expressed as mean with ± SD and were compared using the *t*‐test and Mann–Whitney *U* test. Categorical variables were expressed as number (percentage) and were compared between groups using Chi‐square or Fisher's exact test as appropriate. A logistic regression model was used to assess predictive factors of 6‐week mortality. Mortality prediction accuracy was assessed using area under the receiver operating characteristic (AUROC) curve. We compared the new model to existing models of MELD, Child–Pugh, and Glasgow Blatchford scores. We used a bootstrapping approach to validate the predictive model.

The final model was validated using cross‐validation technique. The performance of the cross‐validation model and the final predictive model was assessed using confusion matrices to compare predicted outcomes against true outcomes, and by examining diagnostic accuracies using the optimum threshold, that is, highest sensitivity and specificity, according to the ROC of the original predictive model.

## Results

### Study cohort, patient features, and outcomes

From October 2012 to September 2018, we included 1161 consecutive cirrhotic patients with AVB and non‐variceal bleeding admitted to Phramongkutklao and Maharat Nakhon Sri Thammarat Hospitals. After excluding subjects under 18 years of age (*n* = 2), not undergoing esophagogastroduodenoscopy (EGD) (*n* = 5), and those lost to follow‐up within 6 weeks from the initial endoscopic exam (*n* = 10), 713 patients with AVB remained and were included in the study. Among those, 89.5% were male (*n* = 638) and 87.2% currently consumed alcohol (*n* = 622). Flowchart for the study participant is demonstrated in Figure S1. Baseline characteristics of the patients at admission are reported in Table 1. Clinical outcomes of patients during admission are reported in Table 2. The overall 6‐week mortality was 18%. Causes of death were uncontrolled bleeding among 88 patients (12.3%), sepsis among 31 patients (4.4%), and cardiovascular events (heart failure, myocardial infarction) among 7 patients (1%). The mean hospitalization time for patients was 13 days.

**Table 1:** Baseline characteristics of patients at admission

Parameter		Total, *n* (%)	AVB, *n* (%)	NVB, *n* (%)	*P*‐value
Hospital	Phramongkutklao	384 (33.5%)	238 (33.4%)	146 (33.9%)	0.864
	Nakhon Sri Thummarat	760 (66.5%)	475 (66.6%)	285 (66.1%)	
Sex	Male	1014 (88.0%)	638 (89.5%)	376 (87.2%)	0.247
	Female	130 (12.0%)	75 (10.5%)	55 (12.8%)	
Age (years)	Mean ± SD	53.3 ± 12.0	53.3±12.2	53.3 ± 11.6	0.982
Admission date	Weekday	746 (66.0%)	474 (66.5%)	272 (63.1%)	0.246
	Weekend	398 (34.0%)	239 (33.5%)	159 (36.9%)	
Chief complaint	Hematemesis	972 (85.0%)	607 (85.1%)	365 (84.7%)	0.838
	Melena	189 (16.5%)	122 (17.1%)	67 (15.6%)	0.490
	Both	17 (1.5%)	16 (2.2%)	1 (0.2%)	0.006
Time to vasoactive agents (min)	Mean ± SD	64.4 ± 15.9	66.2 ± 16.0	61.6 ± 15.5	<0.001
Time to endoscopy (h)	Mean ± SD	42.7 ± 22.7	43.2 ± 22.6	41.9 ± 22.7	0.357
Child–Pugh Score	A	191 (16.7%)	102 (14.3%)	89 (20.7%)	0.002
	B	802 (70.1%)	502 (70.4%)	300 (69.6%)	
	C	151 (13.2%)	109 (15.3%)	42 (9.7%)	
MELD score	<18	858 (75%)	517 (72.5%)	341 (79.1%)	0.012
	≥18	286 (25%)	196 (27.5%)	90 (20.9%)	
	Mean ± SD	16.5 ± 4.0	16.6 ± 4.1	16.4 ± 3.9	0.427
Etiology of cirrhosis	Alcohol	933 (81.6%)	589 (82.6%)	344 (79.8%)	0.528
	Hepatitis B	40 (3.4%)	20 (2.8%)	20 (4.6%)	
	Hepatitis C	131 (11.5%)	80 (11.2%)	51 (11.8%)	
	Others	40 (3.5%)	24 (3.5%)	16 (3.8%)	
Alcohol abuse	Current use	1002 (87.6%)	622 (87.2%)	380 (88.2%)	0.644
	None	142 (12.4%)	91 (12.8%)	51 (11.8%)	
Mean arterial pressure at presentation (mmHg)	Shock	858 (75.0%)	517 (72.5%)	341(79.1%)	0.012
	>50	286 (25.0%)	196 (27.5%)	90(20.9%)	
	Mean ± SD	65.5 ± 13.9	63.9 ± 14.8	68 ± 11.8	<0.001
Heart rate (beat/min)	Mean ± SD	82.5 ± 12.9	83 ± 13.2	81.7 ± 12.6	0.093
Hemoglobin (g/dL)	Mean ± SD	9.8 ± 1.8	9.7 ± 1.9	9.9 ± 1.7	0.014
Platelet count (per L)	Mean ± SD	87 190 ± 38 378	86 727.9 ± 39 477.6	87 955.9 ± 36 520.2	0.600
Glasgow‐Blatchford score	0–1	300 (26.2%)	182 (25.5%)	118 (27.4%)	<0.001
	2–5	604 (52.8%)	346 (48.5%)	258 (59.9%)	
	>5	240 (21.0%)	185 (26.0%)	55 (12.8%)	
Underlying disease	Hypertension	162 (14.2%)	103 (14.5%)	59 (13.7%)	0.722
	Diabetes	43 (3.7%)	36 (5.1%)	7 (1.6)	0.003
	Hepatocellular carcinoma	125 (10.9%)	86 (12.1%)	39 (9.1%)	0.113
	Cholangiocarcinoma	68 (5.9%)	44 (6.2%)	24 (5.6%)	0.676
	Other malignancy	42 (3.6%)	25 (3.5%)	17 (3.9%)	0.703

AVB, acute variceal bleeding; MELD, model for end‐stage liver disease; NVB, non‐variceal bleeding.

**Table 2:** Clinical outcomes of patients during admission

Parameter		Total, *n* (%)	AVB, *n* (%)	NVB, *n* (%)	*P*‐value
Previous bleeding	Esophageal varices	520 (45.5%)	300 (42.1%)	220 (51.0%)	0.003
	Peptic ulcer	240 (21.0%)	143 (20.1%)	97 (22.5%)	0.423
	Gastric varices	49 (4.3%)	47 (6.6%)	2 (0.5%)	<0.001
Endoscopic finding: Esophageal varices with	Active bleed/white nipple	161 (14.1%)	161 (22.6%)	0 (0%)	0.016
	Gastric varices	22 (1.8%)	22 (3.1%)	0 (0%)	<0.001
	Erosive gastroduodenitis	379 (33.2%)	124 (17.4%)	255 (59.2%)	<0.001
	Peptic ulcer	486 (42.5%)	55 (7.7%)	431 (100%)	<0.001
Controlled bleeding rate		1009 (88.2%)	600 (84.1%)	34 (50.9%)	0.001
Rebleeding rate	5 days	155 (13.6%)	101 (14.2%)	54 (12.5%)	0.001
	6 weeks	244 (21.3%)	180 (25.3%)	64 (14.9%)	<0.001
Mortality	5 days	110 (9.6%)	109 (15.3%)	1 (0.2%)	<0.001
	6 weeks	141 (12.3%)	128 (18.0%)	12 (2.9%)	<0.001
Causes of dead	Bleeding	97 (8.5%)	88 (12.3%)	9 (2.09%)	<0.001
	Sepsis	34 (3.0%)	31 (4.4%)	3 (0.7%)	<0.001
	Cardiovascular	7 (1%)	7 (1.0%)	0 (0%)	0.049
Packed red cell transfusion (unit)	Mean ± SD	2.3 ± 1.3	2.6 ± 1.3	1.9 ± 1.2	<0.001

AVB, acute variceal bleeding; NVB, non‐variceal bleeding.

### Predictive factors of 5‐day and 6‐week mortality

We assessed both 5‐day and 6‐week mortality after AVB. All significant univariate variables were chosen for multivariate analysis with objective variables available on admission to develop the best data‐driven model. We found that MELD score ≥18, shock, and endoscopic findings of esophageal varices with high‐risk stigmata (active bleed or white nipple sign) increased the risk of mortality (Table 3).

**Table 3:** Factors associated with 5‐day and 6‐week mortality in multivariate analysis

Risk factor	5‐Day mortality		6‐Week mortality	
	HR (95% CI)	*P*‐value	HR (95% CI)	*P*‐value
Shock	12.25 (7.09–21.16)	<0.001	12.91 (7.95–20.97)	<0.001
Active bleeding or whit nipple sign	7.72 (4.66–12.79)	<0.001	4.43 (2.97–6.62)	<0.001
MELD ≥ 18	1.55 (1.03–2.35)	0.037	2.05 (1.38–3.05)	<0.001

CI, confidence interval; HR, hazard ratio; MELD, model for end‐stage liver disease.

### Model generation and performance of 6‐week mortality

**Figure 1:**
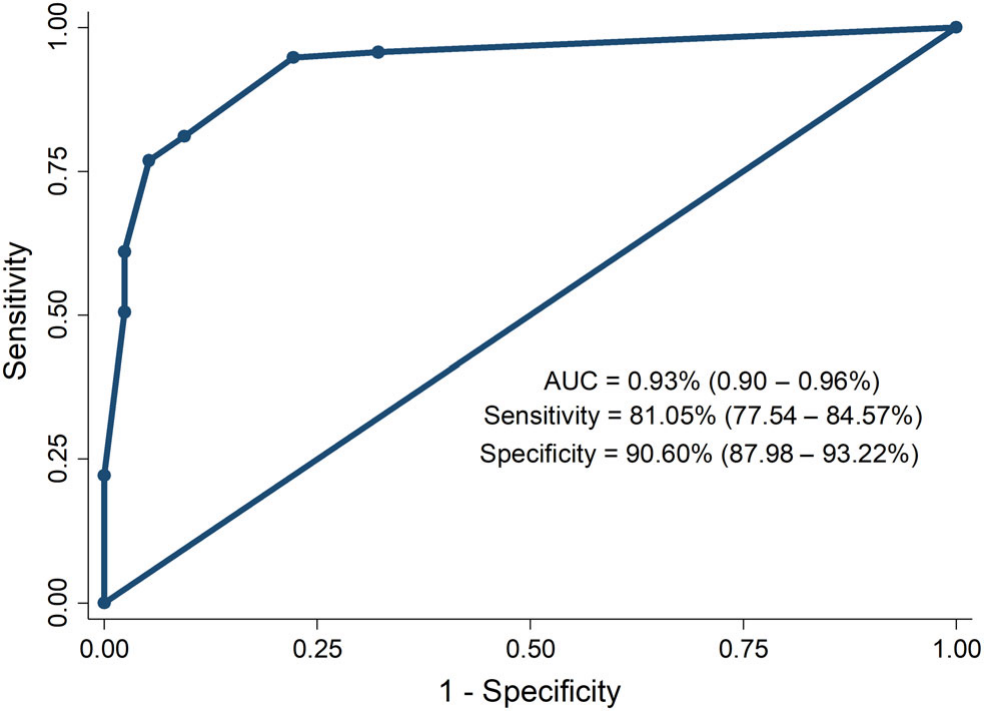
Model to predict 6‐week mortality in patients with acute variceal bleeding—Derivation cohort. AUC, area under the receiver operating characteristic.

**Figure 2:**
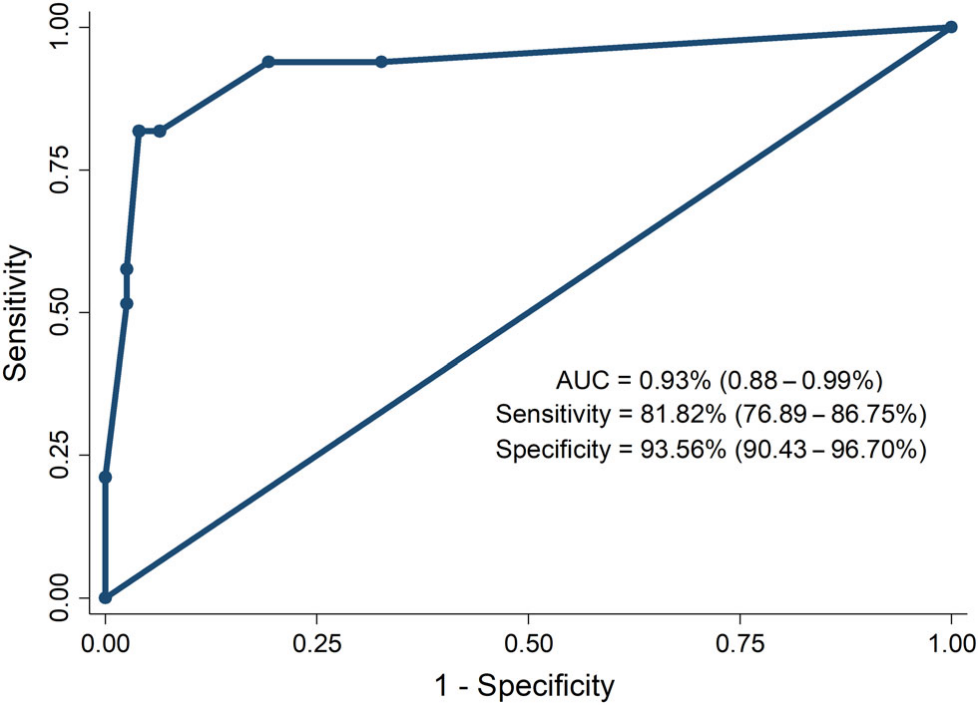
Model to predict 6‐week mortality in patients with acute variceal bleeding—Validation cohort.

The newly developed prognostic model to predict 6‐week mortality after AVB episode, obtained with logistic regression analysis, is presented in Table 3. This model was developed from independent prognostic factors using multivariate analysis and calculated using the equation (1.5 × MELD score ≥18) + (3.5 × shock) + (1 × time to endoscopy < 24 h) (Table 4). Discriminatory performance was evaluated by analyzing the ROC curves. Our model with cutoff value ≥4 (Figs 1, 2) demonstrated the best predictive accuracy among all prognostic variables (AUC, 0.93; 95% confidence interval [CI], 0.9–0.96), sensitivity (81.05%; 95% CI, 77.5–84.5) and specificity (90.6%; 95% CI, 87.98–93.22). Cross‐validation datasets were used to evaluate the validity of our model. Little difference was found in predictive accuracy between the final model and the cross‐validation model (AUC, 0.93; 95% CI, 0.88–0.99).

**Table 4:** Multivariate analysis for 6‐week mortality

Risk factor	6‐Week mortality		Multivariate analysis		Risk score
	No	Yes	OR (95% CI)	*P*‐value	
Shock	20 (21.5%)	73 (78.5%)	48.98 (23.78–100.86)	<0.001	3.5
MELD ≥ 18	74 (53.2%)	65 (46.8%)	6.09 (3.01–12.33)	<0.001	1.5
Time‐endoscopy < 24 h	54 (60.0%)	36 (40.0%)	2.91 (1.34‐6.31)	0.007	1

Developing model to predict mortality		
AVB (*n* = 713)	Mortality in 6‐week	
	No	Yes
Derivation cohort (*n* = 478)	383 (80.13)	95 (19.87)
Validation cohort (*n* = 235)	202 (85.96)	33 (14.04)

This model was developed from independent prognostic factors using multivariate analysis and calculated using the equation (3.5 × shock) + (1.5 × MELD score ≥ 18) + (1 × Time‐endoscopy < 24 h)		
Cut of point ≥ 4	Derivation cohort	Validation cohort
AUC	0.93% (0.90–0.96%)	0.93% (0.88–0.99%)
Sensitivity	81.05% (77.54–84.57%)	81.82% (76.89–86.75%)
Specificity	90.60% (87.98–93.22%)	93.56% (90.43–96.70%)

AVB, acute variceal bleeding; AUC, area under the receiver operating characteristic; CI, confidence interval; MELD, model for end‐stage liver disease; OR, odds ratio.

### Risk factors associated with rebleeding in AVB

Rebleeding of AVB was defined as a sudden clinical deterioration with a concomitant increase of gastrointestinal hemorrhage after controlled variceal bleeding by initial endoscopy. We assessed both 5‐day and 6‐week rebleeding. After multivariate analysis, the covariates associated (*P* < 0.05) with a rebleeding within 5 days and 6 weeks after initial endoscopy are summarized in Table 5. MELD score ≥18 was associated only with 6‐week rebleeding.

**Table 5:** Factors associated with 5‐day and 6‐week rebleeding in multivariate analysis

Risk factor	5‐Day rebleeding		6‐Week rebleeding	
	HR (95% CI)	*P*‐value	HR (95% CI)	*P*‐value
Glasgow Blatchford score ≥ 6	3.68 (2.08–6.51)	<0.001	2.25 (1.33–3.81)	0.002
HxUGIB	2.27 (1.53–3.38)	<0.001	2.26 (1.56–3.27)	<0.001
Shock	2.32 (1.30–4.15)	0.004	2.15 (1.27–3.64)	0.004
Weekend	1.56 (1.08–2.27)	0.018	1.44 (1.04–1.99)	0.028
Time‐endoscopy <24 h	1.46 (0.98–2.17)	0.062	1.21 (0.84–1.74)	0.312
MELD ≥ 18	—	—	2.22 (1.54–3.19)	<0.001
NSAIDs	—	—	3.96 (2.33–6.72)	<0.001
Current alcohol abuse	—	—	1.92 (1.06–3.48)	0.031
PMK Hospital	—	—	1.49 (1.06–2.09)	0.023

CI, confidence interval; HR, hazard ratio; MELD, model for end‐stage liver disease; NSAID, Nonsteroidal anti‐inflammatory drug; PMK, Phramongkutklao.

## Discussion

The results of our study show that combined MELD score, MAP, and high‐risk stigmata sign during endoscopy could identify, and with improved accuracy to predict 6‐week mortality after cirrhosis present with AVB. Furthermore, cross‐validation analysis for internal accuracy of the model demonstrated it is highly calibrated with a strong ability to discriminate at‐risk features of cirrhotic patients with AVB during admission. This study was conducted on our large cohorts with a consecutive cirrhotic patient sample with a diagnosis of AVB over a 5‐year period. Using this new model, marked improvement in identifying cirrhotic patients at risk of AVB compared with related studies.^12^

The model seemed to have better performance characteristics as compared with the MELD score alone developed to predict outcomes of cirrhosis. Various risk‐scoring systems have been developed to discriminate patients with UGIB in high‐ and low‐risk groups. In fact, most scoring systems including Glasgow‐Blatchford score developed and validated peptic ulcer bleeding. The use of these scoring systems in cirrhotic patients with AVB may be confounded by the severity of liver disease and poor at predicting clinical outcome within this group.^6^ Patients with cirrhosis together with variceal bleeding may have been influenced by history of liver dysfunction. Childs–Pugh score, MELD score, and the hepatic venous pressure gradient are recognized models to predict outcome among patients with cirrhosis.^8^, ^13^, ^14^ However, important variables at pre‐endoscopy and during endoscopic examination were excluded. Adding those variables including MAP and high‐risk stigmata during endoscopy in previous models increased the ability to discriminate features of high‐risk mortality of cirrhotic patients with AVB. Recalibrating the MELD score was important and needed. Adding variables to the MELD score can develop a new MELD calibration to predict the mortality of cirrhotic patients within 6 weeks of presentation with AVB.^15^ Several models have been developed to predict prognosis and identify high‐risk patients after AVB episode. Some models have used initial laboratory‐based equations to predict 6‐week mortality but the equations are complex and may not be applicable to identify risk at the initial management.^12^

In addition, we found significant correlations between the MELD score and rebleeding. Rebleeding is associated with mortality as high as the initial bleeding and needs prevention. Our study found that patients with advanced liver disease were also at risk for rebleeding after surviving their first bleeding. Managing high‐risk patients should be followed, which must include optimal resuscitation, early administration of vasoactive agent and antibiotic prophylaxis, intensive care unit admission, and prompt endoscopic hemostatic intervention.^1^ To achieve hemostasis, optimal endoscopic treatment should be performed as soon as patients gain hemodynamic stability. Since emergent EGD is unavailable at our institutions on a 24/7 basis with an on‐call endoscopist and support staff, we observed the weekend effect of cirrhotic patients with AVB increased risk of rebleeding. The timing of endoscopic intervention might be a factor influencing morbidity of cirrhotic patients with AVB. Although overall inhospital mortality was similar between weekday and weekend admission, weekend patients may be less likely to undergo endoscopy within the first few days of admission. The weekend effect in our study was similar to that in the US Nationwide Inpatient Sample database and meta‐analysis result. Admission on the weekend has no difference in mortality when compared with weekday admission.^16^, ^17^ Several potential explanations may be applied to this result. First, a weekend effect may not apply to cirrhotic patients with AVB because natural history of variceal bleeding is more complicated than non‐variceal bleeding. Patients with variceal bleeding often had more risk factors and comorbidities, such as presence of liver cirrhosis, severe portal hypertension, hepatocellular carcinoma, and renal and circulatory dysfunction. These explanations applied at time of admission played only a small role in AVB mortality. Another explanation for this finding was that assessing the influence of weekend admission on mortality may have been underestimated because patients admitted during a weekend may consider receiving endoscopy on a weekday.

In our study, the time of endoscopic intervention <12 h increases the risk of 6‐week mortality. Potential explanation may be applied to this finding due to patients received prompt endoscopy within 24 h was sicker than elective endoscopy. Emergent EGD is not available at our institutions on a 24/7 basis with an on‐call endoscopist and support staff. Patients who were stable enough for medical treatment especially admitted during a weekend may consider receiving endoscopy on a weekday.

Our study encountered some limitations. First, using an administrative database has influenced its reliability for research purposes and lack of documentation regarding the quality of the collected data. Second, our study result was linked to restricted study populations in tertiary referral hospitals. Third, our data have resource‐limited facility for emergency EGD setting; the average time to endoscopy in our data sets is too long, which is the 5‐day mortality could be significantly affected by the delay. This information may not reflect the practice of other facilities in the medical field. Lastly, we performed only internal validation of our new model.

In summary, among patients with cirrhosis and AVB, our new model offered an accurate prognostic prediction with simple variables available early after admission. The new model could be employed to identify high‐risk patients who might benefit from greater attention and more aggressive treatments. Patients with higher MELD score should be closely monitored due to the higher probability of 6‐week rebleeding. Future studies to further validate the efficacy of our new model are needed to confirm its performance described retrospectively in databases.

## Supporting information


**Figure S1.** Flowchart for the study participant.
