# Supplementary figures and images for: Development and validation of prognostic model to predict mortality among cirrhotic patients with acute variceal bleeding: A retrospective study
Source: JGH Open. 2021 May 5;5(6):658–63. doi: 10.1002/jgh3.12550 (PMC8171152; doi:10.1002/jgh3.12550)

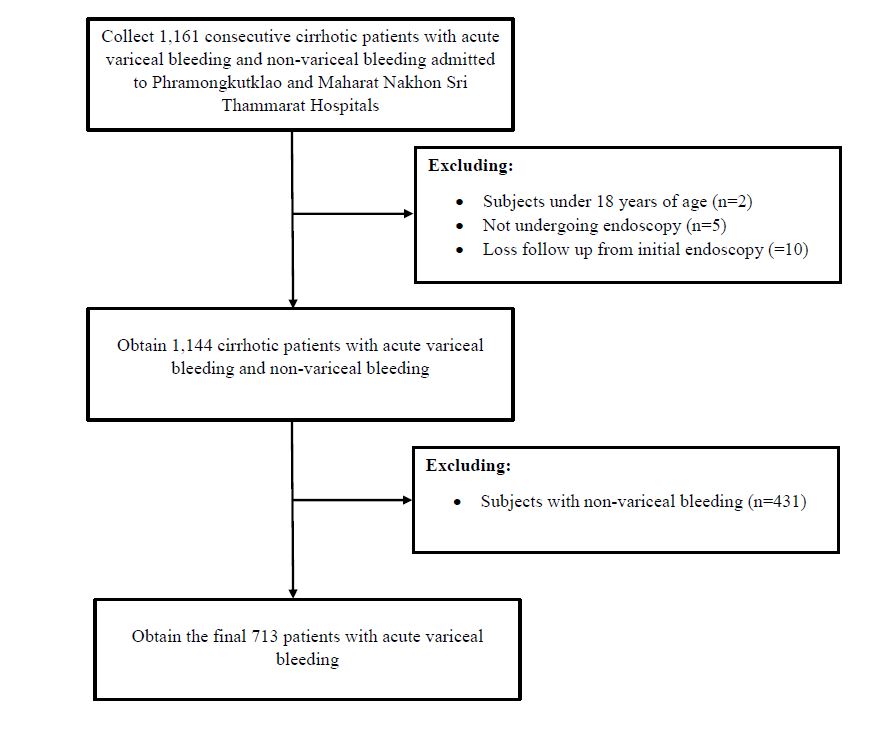

Supplement: Supplementary file 1 — Figure S1. Flowchart for the study participant. [file JGH3-5-658-s001.JPG]
